# School Bullying Is Not a Conflict: The Interplay between Conflict Management Styles, Bullying Victimization and Psychological School Adjustment

**DOI:** 10.3390/ijerph191811809

**Published:** 2022-09-19

**Authors:** Christoph Burger

**Affiliations:** 1 Department of Developmental and Educational Psychology, Faculty of Psychology, University of Vienna, Liebiggasse 5, A-1010 Vienna, Austria; christoph.burger@univie.ac.at; Tel.: +43-1-2931532; 2 Department of Cognition, Emotion, and Methods in Psychology, Faculty of Psychology, University of Vienna, Liebiggasse 5, A-1010 Vienna, Austria; 3 Department of Psychology and Psychodynamics, Karl Landsteiner University for Health Sciences, Dr.-Karl-Dorrek-Straße 30, A-3500 Krems, Austria

**Keywords:** bullying, school violence, victimization, bully-victims, conflict management, psychological adjustment, psychological maladjustment, integrating

## Abstract

It has been argued that adaptive conflict management styles may protect students against bullying victimization and against negative effects of ongoing victimization on psychological school adjustment. Moreover, maladaptive conflict management styles may lead to victimization or intensify negative effects of victimization on school adjustment. Mediation and moderation models were computed to test these effects. Furthermore, a person-oriented approach compared noninvolved students, victims, and bully-victims regarding conflict management styles and school adjustment. A total of 172 individuals (77.2% female, mean age: 22.7 years) completed a retrospective online questionnaire about conflict management styles, bullying victimization and school adjustment during their school years. In the mediation model, conflict management styles were not associated with victimization, but there was a positive direct effect of the integrating style on school adjustment. In the moderation model, the integrating style moderated the negative effect of victimization on school adjustment but did not buffer against the negative effects when victimization was high. Person-oriented comparisons showed that victims used the obliging style more often than bully-victims. Furthermore, victims and bully-victims showed lower school adjustment than noninvolved students. Overall, results corroborate the view that school bullying is qualitatively different from normal peer conflicts. Implications for researchers, policymakers, school principals and teachers are discussed.

## 1. Introduction

It has been repeatedly suggested that conflict management skills might be an effective means of tackling bullying victimization [1,2,3] and its harmful effects on psychological adjustment. However, others disagree, pointing out that while conflict management skills might help resolve everyday conflicts among peers, they are not useful for resolving actual bullying cases. This is because peer conflicts are fundamentally different from bullying victimization cases [4]. Typical everyday conflicts among peers of equal status manifest in situations of incompatibility, disagreement or dissonance [5] where both parties can make their interests count, while bullying victimization is understood as systemic aggressive behavior repeatedly inflicted on a victim over an extended period of time by one or more perpetrators with a clear power advantage and the intent to harm [6]. Surprisingly, there is hardly any research that empirically examined whether different conflict management styles can avert or promote victimization or whether the negative effect of victimization can be reduced or amplified through the use of conflict management styles. Moreover, previous research examining conflict management styles in bullying situations has focused exclusively on workplace bullying among adults, while there is only a single study examining these contentious questions in relation to bullying among students. The present study aims to fill this knowledge gap and shed light on the relationships between conflict management, bullying victimization, and psychological adjustment in the school context. It is important to have a clear answer to these questions so that anti-bullying programs and teacher trainings have clarity on how to address bullying more effectively [7].

During their school years, students are constantly confronted with a changing set of social and academic challenges to which they have to continually adjust [8]. It is the time when the influence of their parents diminishes and interaction with peers and classmates becomes more significant. These interactions provide opportunities to build social skills and new friendships, but also inevitably lead to everyday interpersonal conflicts between peers. These conflicts are an integral part of youth development and how students manage such conflicts can promote or impair their academic adjustment, personal well-being, health, and moral and emotional growth [9,10,11].

### 1.1. The Dual Concern Model of Conflict Management Styles

**Figure 1:**
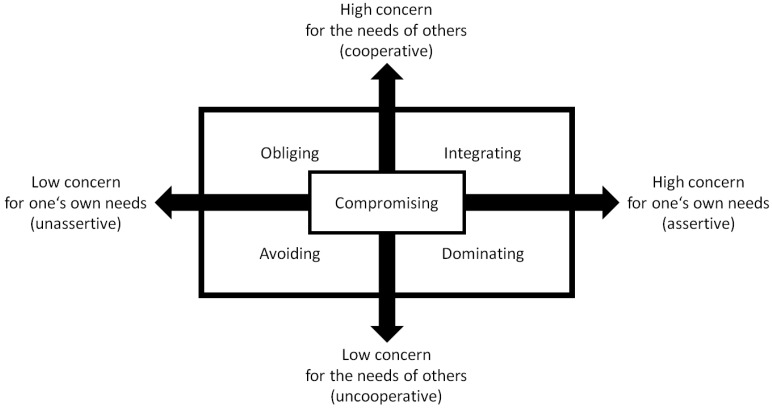
A schematic visualization of the two continua generating a fourfold table of conflict management styles.

In the study of conflict management, two continua are usually distinguished that motivate individuals (dual concern model): concern for self, which aims at satisfying one’s own needs, and concern for others, which aims at satisfying the needs of others [12]. The two continua generate a fourfold table with four distinct strategies (see Figure 1), with each continuum having either a low or a high level: (i) integrating, (ii) obliging, (iii) dominating, and (iv) avoiding. A fifth conflict management strategy emerges when both continua have medium levels: (v) compromising. Conflict management styles can be regarded as relatively stable traits [13] that may have been partially acquired from parents [14].

The *integrating style* is composed of a high concern for both oneself and for others and aims at fully satisfying the interests of both conflict parties [12]. This style requires great flexibility and the willingness of both parties to look at the problem situation from different perspectives. Individuals using it tend to show no fear of self-assertion [15], but they do not aim to impose their own needs on others. They are also willing to cooperate with others, listen to the others’ point of view, exchange information, and explore in detail the differences in their respective needs in order to eventually find alternative solutions that might effectively and fully resolve the needs of both parties [12].

The *dominating style* also involves caring deeply about oneself but having only little to no concern for the needs of others. This style leads to forcing behavior that imposes one’s own goals on others while ignoring or actively disregarding the needs of others [12]. It is usually considered an aggressive strategy characterized by a willingness to defy social norms [15], a high degree of assertiveness, and a marked lack of cooperation [11].

The *obliging style* is a combination of low concern for self and high concern for others and seeks to resolve differences between two parties by satisfying the needs of the other at the expense of one’s own needs. This style is usually characterized by a lack of self-assertion [15] and high agreeableness [16], which leads to subordinate, submissive, and obedient behavior [12]. Individuals using this style tend to play down differences to emphasize communalities with others [11], and believe that matters in their lives are beyond their control and influenced by powerful others [15].

The *avoiding style* represents caring little about one’s own needs and little about the needs of others [12]. Individuals using this style accept severely restricting their own scope of action and thus also the fulfillment of their own needs in order to not find themselves in conflict with others [12]. Individuals using this style score high on neuroticism [16] and low on self-assertion [15], are less cooperative and tend to ignore, evade or avoid conflict situations or conflict parties [17].

Finally, the *compromising style* consists of taking care of one’s own needs and those of the other to an intermediate degree [12]. This style aims to find the middle ground where both parties give up some of their personal subgoals and needs in order to reach a mutually acceptable decision. This includes making mutual concessions and seeking to satisfy, moderately and partially, the interests of all parties to the conflict [11].

### 1.2. Conflict Management Styles and Psychological School Adjustment

The outcome variable of interest in the present study is psychological school adjustment in students. Studies addressing the relationship between conflict management styles and psychological adjustment are, however, scarce and most research has been conducted in the organizational or workplace context. Those few studies that relate to schools have been targeted almost exclusively towards adults such as educators and school managers [18,19,20,21,22] (see Wang et al. [23] for an exception examining the effect of conflict management styles on school adjustment in a sample of urban Chinese pre-adolescents). It is, however, highly important to explore this link also for students as psychological adjustment to the social and academic demands in the school setting is a major task for them [24], and it is plausible that the way they manage conflicts might make a difference in this regard. Meeting the demands of school is a challenging endeavor because, in addition to managing increasingly difficult academic tasks and negotiating complex interpersonal relationships and disagreements with classmates and teachers, students also face developmental challenges as their bodies and identities are constantly changing in the process of growing up [8,25]. As conflict in the classroom is an inevitable part of reality, appropriate strategies are needed to manage it in a way that realizes the potential benefits of conflict and minimizes or eliminates its harmful effects [11].

The *integrating and compromising styles* are cooperative solution-oriented strategies, associated with higher levels of emotional intelligence [18], considered to be relatively effective and appropriate by peers [26], related to higher school adjustment [23], and generally more likely to lead to positive organizational and individual outcomes [27]. If both parties succeed in resolving a conflict constructively and to their mutual benefit, this can lead to higher self-esteem and greater self-efficacy in dealing with such situations. This increased sense of control, combined with a sense of accomplishment, can in turn improve health and well-being [28,29].

The *dominating style* might lead to positive short-term outcomes from the dominant person’s perspective. As a destructive and uncooperative strategy, it is however considered as inappropriate by peers [26] and in the long term it might deteriorate interpersonal relationships. It might have negative effects on future social interactions (e.g., heightened levels of tension and anxiety [28,30]) and result in conflict escalation [27] and undesirable relationship outcomes [31]. Whereas some studies showed that less cooperative maladaptive styles were associated with fighting and low academic achievement in adolescents [32] others found that they were not necessarily negatively associated with school adjustment [23].

The *obliging and avoiding styles* are passive strategies that may provide benefits by minimizing the escalation of negative emotionality in critical moments of a conflict [33]. They might however have negative long-term consequences for self-esteem, self-efficacy and school adjustment [23,30]. The reason might be that the conflict either persists or is resolved at one’s own expense, leading to strain and exhaustion as their users disregard their own desires [34]. It is also known that avoidance promotes rather than reduces existing anxiety, potentially leading to loneliness and poor school adjustment [35]. Repeatedly suppressing and holding back emotions (e.g., anger) as a response in conflicts have also been linked to adverse health outcomes [28,36].

Generally, when considering their application in normal conflicts, integrating and compromising are considered adaptive strategies, whereas dominating, avoiding, and obliging are considered maladaptive strategies because they are likely to lead to unfavorable psychological outcomes [33]. It has to be noted that these styles represent self-reported predispositions for handling conflicts [37]. It should be evident that actual conflict behavior depends heavily on the interaction with the conflict partner and other contextual factors [27] and that any behavior might not necessarily lead to its intended effect.

### 1.3. Bullying Victimization

There are at least three different views of conceptualizing bullying. First, the *bullying as normal conflict* view regards bullying as a normal conflict [38,39,40,41] and sometimes trivializes it as a universal childhood experience that builds students’ character [42]. Second, the *bullying as escalated conflict* view regards bullying as a process that begins as a subtle conflict among equals but escalates due to the inappropriate application of conflict management [1,43,44]. Third, the *bullying being distinct from conflict* view regards bullying as qualitatively different from conflict [4,6,45]. This view particularly emphasizes two aspects of bullying that distinguishes it from conflict: (a) the intention of the perpetrators which do not aim to resolve a mutual disagreement [46] but aim to hurt the victims actively and intentionally [6,44] and (b) the asymmetrical constellation in which perpetrators are in control of the victims due to a clear power advantage [6,47]. This power imbalance exists a priori and is therefore present right from the beginning of the bullying victimization. Perpetrators specifically select victims based on this imbalance and choose victims that are less powerful physically or in numbers or that have lower social status (e.g., being a member of a minority, being overweight, etc.) [48].

A large number of studies have established that bullying victimization can have devastating short- and long-term effects on students’ health and wellbeing but also on their general functioning [49] and more specific on their psychological school adjustment [50]. Victims of bullying are more likely to harbor more negative attitudes toward school [51] because they feel unsafe [52], show poor school performance [53] and are more likely to be absent from school [54]. Research has shown that so-called bully-victims, that is victims that are also perpetrators at the same time, tend to be at an even higher risk of psychological adjustment problems than pure victims. Bully-victims also report receiving little support from teachers and generally have higher rates of school absenteeism than victims [55].

### 1.4. Empirical Findings on the Effects of Conflict Management Styles on Bullying Victimization

Empirical research on the effect of conflict management styles on bullying victimization has yielded mixed results. Previous studies showed that workplaces or teams where a dominating conflict management style was used had higher levels of bullying victimization and where problem-solving styles (i.e., integrating, compromising) were used had lower rates of bullying victimization [56,57,58]. A further study in the workplace context revealed that individuals with a lower power position who use the integrating style were more likely to be victimized [46]. Other studies, however, showed that individuals when confronted with bullying might not be able to use their preferred conflict management styles [1,59], thus their preferred behavior in conflicts might not be predictive of their actual behavior in bullying situations. This was corroborated by a longitudinal study examining these associations on an individual level which found that conflict management styles were not associated with bullying victimization in the workplace context (but high levels of the dominating and low levels of the integrating styles were related to bullying perpetration [44]).

Although the focus of this article is on the school setting, there was no choice but to draw on findings from the workplace setting in the above paragraph because all of the available research of conflict management styles on bullying victimization has been conducted in the workplace context. A notable problem with most workplace studies is that both predictors (i.e., conflict management styles) and outcome variables (i.e., workplace bullying) were collected at an aggregated level (e.g., at the team or organizational level) rather than at the individual level. This provides an undifferentiated view, as averaging across victims and perpetrators conceals potentially important differences between these two groups in their use of conflict management styles. The only study that differentiated between victims and perpetrators found [44] that conflict management styles were only predictive of bullying for perpetrators but not for victims. This corroborates the view that in bullying situations perpetrators and victims play distinct roles [46] and that future research examining the effect of conflict management style use by victims should not aggregate predictors and outcomes across groups of people. In sum, since workplace and school contexts differ in key aspects, the question arises to what extent the findings from the workplace context are relevant to the school context. This shows the clear need for research in the school context that addresses these issues at an individual level.

### 1.5. Empirical Findings on the Moderating Effects of Conflict Management Styles on the Negative Effect of Bullying Victimization on School Adjustment

Even if conflict management styles cannot prevent victimization, it could be that they help students regulate the negative effects of ongoing victimization on school adjustment as a means of better dealing or coping with their perpetrator in the victimization situation. Adaptive conflict management styles might reduce or eliminate the negative effect of victimization on school adjustment, while maladaptive conflict management styles might exacerbate it. Thus, it makes sense to examine, as a second line of inquiry, whether the use of different conflict management styles moderates the negative effect of victimization on school adjustment.

There is only one study that investigated this hypothesis in a school context. This study was conducted with a sample of rural Chinese students and supported this moderation hypothesis. Solution-oriented styles (e.g., integrating, compromising) attenuated the negative effect of victimization on school adjustment, whereas non-confrontational styles (e.g., avoiding, obliging) exacerbated it [23]. There are also some results available from studies carried out in the workplace context. A German study corroborated the finding of the Chinese study, as more avoidant/unassertive conflict management styles exacerbated the negative effects on psychological adjustment in victims of workplace bullying [60]. Further studies in the workplace context showed that the use of cooperative strategies in which the victim shares information with the perpetrator (e.g., integrating, compromising) were not only ineffective in stopping the victimization, but increased negative personal outcomes for the victims [1,61,62] as perpetrators tend to use information against the victim. Other studies showed that victims of bullying may not be able to use their preferred conflict management styles in bullying situations and therefore might resort to base levels of ineffective behaviors [1,59].

In summary, the only study conducted in a school context showed that different conflict management styles can reduce or increase the negative effects of bullying victimization. However, since the sample consisted of rural Chinese pre-adolescents, these results might not be generalizable to more individualistic Western societies [63]. In addition, the results of studies on workplace bullying are mixed regarding the moderation hypothesis of conflict management styles, with some confirming the moderation hypothesis and others suggesting that in bullying situations, victims may not be able to use their preferred conflict management styles because perpetrators are in control of the situation. Since it is unclear to what extent the findings from rural China or from the workplace context can be applied to the general school context in Western societies, there is great need for future studies to clarify this.

### 1.6. The Development of Research Hypotheses from the Three Views on Conceptualizing Bullying

Because the empirical findings to date are either mixed or inapplicable to the school context, they are not suitable for formulating explicit research hypotheses. In the present study, therefore, the three views of conceptualizing bullying introduced in Section 1.3 are used to formulate research hypotheses regarding the roles that conflict management styles play in the context of bullying victimization and psychological adjustment in the school setting. Depending on the results of the present study, conclusions can be drawn about the validity of the respective conceptual views on bullying.

First, if the *bullying as normal conflict* view were true, conflict management styles would be as effective in bullying situations as they are in ordinary conflicts caused by mutual disagreements among peers of equal strength. Thus, adaptive conflict management styles aimed at constructive problem solving, such as compromising and integrating, should result in less victimization, and maladaptive strategies, such as avoiding, dominating and obliging, should lead to more victimization in the long run. Similarly, adaptive conflict management styles should also diminish the negative effect of bullying on school adjustment while maladaptive styles should increase it.

Second, the *bullying as escalated conflict* view explains the prevention or emergence of bullying by how conflict management styles avoid or reinforce escalation in critical conflict situations [64]. Bullying victimization would be expected to be prevented by de-escalating conflict management styles that seek to downplay the conflict or constructively resolve the issues behind it (avoiding, obliging, integrating, compromising) and caused by escalating styles that attack the other or force the other to accept a particular solution (dominating) [43]. The dominating style risks escalation through retaliation [65] and is likely to intensify the conflict because it defines it as a win-lose situation [46]. Similar predictions can be made about the abovementioned styles’ reduction or intensification of the negative effects of bullying victimization on school adjustment.

Third, the *bullying being distinct from conflict* view emphasizes the importance of the perpetrators’ a priori power advantage and malicious intention to harm the victim (repetition is not the focus because conflicts can also have multiple episodes). Because of this power disadvantage, victims are completely dependent on the perpetrators’ will in all attempts to manage the conflict. Since the will of the perpetrators is not to resolve a conflict, but to harm the victims, the victims are very likely not able to use the strategies they usually prefer in normal conflicts. The integrating style requires an open exchange of ideas that is most likely not provided by the perpetrators [27]. The compromising style also requires offenders to be willing to forego full enforcement of their goals, which they most likely will not accept because of their power advantage [46]. The avoiding style requires that perpetrators give victims some breathing room; however, since perpetrators aim to harm the victims and victims have to be present at school, perpetrators may seek them out at any time and at their convenience. The avoiding style may however be possible to some degree if victims are truant from school [54]. Attempts by victims to use the dominating style are likely to be immediately and successfully stopped by perpetrators because of their power advantage. Victims might also refrain from using the dominating style in the first place because of fear of retaliation [65]. The only conflict management strategy that victims may be able to employ is the obliging style [46]. Due to the power advantage of the perpetrators, victims might not have much choice but to obey the orders of the perpetrators if they do not want to face massive retaliation. Overall, because the power asymmetry severely restricts the victims in implementing their preferred conflict management styles, this view anticipates that the conflict management styles will play little to no role in both predicting victimization or regulating the negative effects of victimization on school adjustment.

These three views on bullying can also be used to hypothesize different results when comparing bullying-related groups (i.e., victims, bully-victims and noninvolved individuals). The *bullying as normal conflict* view would predict both victims and bully-victims to use lower levels of adaptive and higher levels of maladaptive conflict management styles. Regarding maladaptive styles, bully-victims might use higher levels of dominating and lower levels of obliging than victims. The *bullying as escalated conflict* view would explain victimization by individuals using too few de-escalating conflict management styles (which might be expected from victims) and too many escalating conflict management styles (which might be expected from bully-victims). Finally, the *bullying being distinct from conflict* view would expect no differences between noninvolved individuals and both victims and bully-victims with the only exception that victims might be more submissive and might offer less resistance to obey the perpetrator than bully-victims.

### 1.7. Short-Term Retrospective Measurement of Bullying-Related Behavior

Surveying underage students regarding current bullying victimization may not only result in them not answering honestly because they are in the center of the ongoing incident [66] but may also raise ethical concerns [67,68]. Retrospective surveys of individuals who are of legal age and already out of school could be one possible way out of these issues. Although retrospective data collection has the possible disadvantage of recall bias, the negative effects of this bias should be limited if the sample is chosen so that the school years were not so long ago. After all, bullying incidents have a high personal impact and should not be easily forgotten within years (for a more detailed discussion of this survey method, see Burger and Bachmann [50]).

### 1.8. Current Study

Thus far, surprisingly few studies have investigated whether the use of different conflict management styles might protect students either by inhibiting bullying victimization from happening, or by preventing or diminishing its potential negative effects on psychological school adjustment (see Wang et al. [23] for an exception using a sample of urban Chinese pre-adolescents). Most research has been conducted in workplace settings, and it is unclear whether these results can be generalized to school bullying. Comparing the hypotheses formulated from three distinct views of conceptualizing bullying victimization, this study has three main objectives to advance research in this subject area.

The first goal is to examine whether different conflict management styles are related to psychological school adjustment, and whether these effects are mediated by bullying victimization. In other words, it is examined whether the use of certain conflict management styles may prevent or lead to bullying victimization, and thus, in a further step, may prevent or exacerbate psychological school adjustment. This line of inquiry would aim to gain insights in the effects of conflict management styles as possible antecedents of victimization (i.e., protective vs. risk factors). Such insights could be used in anti-bullying programs to prevent victimization and its harmful consequences. The *bullying as normal conflict* view and the *bullying as escalated conflict* view hypothesize that the occurrence of bullying victimization depends on what conflict management styles victims use. In contrast, the *bullying being distinct from conflict* view hypothesizes that conflict management styles have no effect on bullying victimization, since they are ineffective in such cases or cannot be applied by the victims in the first place due to the malicious intent of the perpetrators and the perpetrators’ non-existent willingness to cooperate [59].

Second, the study examines whether the negative effect of ongoing victimization on school adjustment is moderated by conflict management styles; specifically, whether some conflict management styles reduce or amplify the negative effect of bullying on victims. This direction of research would aim to uncover insights into the effects of conflict management styles as possible negotiating styles during ongoing victimization (i.e., adaptive vs. maladaptive coping of victims with perpetrators). These findings could be used to mitigate harmful consequences in persistent bullying cases on school adjustment. Whereas the *bullying as normal conflict* view would hypothesize a buffering effect of cooperative styles such as integrating and compromising, the *bullying as escalated conflict* view would hypothesize a buffering effect of de-escalating styles such as obliging and avoiding. In contrast, the *bullying being distinct from conflict* view would hypothesize that conflict management styles have no buffering effect on school adjustment in situations of bullying victimization, because victims have no control of the situation due to the power disadvantage and therefore cannot use conflict management styles to improve their situation in school.

Third, the study also takes a person-oriented approach, aiming to compare noninvolved students with victims and bully-victims. Previous research has shown that this approach can yield complementary results as it understands individuals as integrated wholes rather than as single variables [69]. Bully-victims were included because previous research has shown that this group uses more aggressive behavioral strategies and is often more strongly associated with adjustment problems than pure victims [70]. *The bullying as normal conflict* view hypothesizes that both victims and bully-victims use lower levels of integrating and compromising and higher levels of obliging and avoiding. Regarding the dominating style, bully-victims are hypothesized to have higher levels than victims. The *bullying as escalated conflict* view hypothesizes victims to use too few de-escalating styles such as avoiding and obliging and bully-victims to use too many escalating styles such as dominating. Finally, the *bullying being distinct from conflict* view would expect no differences between noninvolved individuals and both victims and bully-victims, with the only exception of victims having higher levels of obliging than bully-victims.

## 2. Materials and Methods

### 2.1. Participants

A minimum age of 18 was set as the inclusion criterion, as participants should have completed their schooling in order to be able to assess them retrospectively in their entirety. The sample comprised 172 individuals (77.2% women) with an average age of 22.7 years (*SD* = 2.29, *min* = 18, *max* = 27). In terms of their professional background, several non-exclusive options could be selected; therefore, the total adds up to more than 100%: 79.7% were university students, 45.9% were employed, 1.7% were unemployed, 1.7% were in military or civilian service, and 0.6% were housewives/househusbands. With respect to their highest educational degree, 63.4% had earned a secondary school leaving certificate (corresponding to university entrance qualification), 29.7% had earned a bachelor’s degree, 4.1% had earned a master’s degree, and 2.9% had earned vocational training (including apprenticeship or vocational school). The sample has been described in more detail elsewhere [71].

### 2.2. Procedure

The online survey was administered at one point in time and was advertised via online social networks. Participants were informed about the key facts of the survey on the first questionnaire page (e.g., inclusion criteria, thematic areas covered, estimated duration, voluntary nature of participation, data protection, anticipated benefits and risks) and were required to provide informed consent to complete the questionnaire. There was no compensation offered to participants. It was clearly indicated to participants on each page of the online questionnaire that all questions pertained to their school years and had to be filled out retrospectively to the best of their personal memory.

### 2.3. Measures

After providing informed consent, participants indicated their gender (0 = female, 1 = male), age, and highest educational degree. Furthermore, the following three constructs were measured. The means, standard deviations, zero-order correlations, and reliabilities of the study variables are reported in the Section 3.

#### 2.3.1. Conflict Management Styles

A German version of the Rahim Organizational Conflict Inventory (ROCI-II, form C; Rahim [27]) by Bilsky and Wülker [15] was adapted to retrospectively measure five different conflict management styles used in conflicts with other classmates during the school years. The inventory consists of 28 5-point Likert items with response options ranging from *strongly disagree* (1) to *strongly agree* (5). The five conflicts management styles measured were labeled integrating (7 items; Cronbach’s α = 0.85), obliging (6 items; α = 0.80), avoiding (6 items; α = 0.84), dominating (5 items; α = 0.84), and compromising (4 items; α = 0.74). Example items are “I try to get to the bottom of a problem together with my classmates in order to find a solution that is satisfactory for everyone”, “I comply with the demands of my classmates”, ”I usually avoid open discussions about differences with my classmates”, “I generally pursue my interests strongly” and “I generally suggest a middle way to get out of deadlocked situations”, respectively.

#### 2.3.2. Psychological School Adjustment

Nine 7-point German Likert items by Burger and Bachmann [50] (based on a scale by Kochenderfer-Ladd [51]) were used to retrospectively capture psychological school adjustment during the school years. Response options ranged from *completely disagree* (1) to *completely agree* (7). Sample items are “When I was in school, I was happy”, and “When I was in school, I worried”. Higher mean scores represent higher levels of adjustment. Reliability was excellent (α = 0.93).

#### 2.3.3. Bullying Victimization

A filter item was utilized to gauge whether study participants were victimized during their school years: “I was harassed, picked on in my class”. The response options were *No, that does not apply to me* (1), *Yes, sometimes* (2), and *Yes, often* (3). If participants answered with options 2 or 3, the 5 item 7-point measure by Burger and Bachmann [50] was used to assess the extent of bullying victimization. Participants retrospectively described how they were exposed to five different types of bullying during their school years, comprising physical, verbal, relational, property, and cyber bullying. Response options ranged from *strongly disagree* (1) to *strongly agree* (7). Before responding to the items, participants read a brief instruction which explained the retrospective bullying measure and made clear it related to their school years. The items were preceded by the question, “How can you describe the harassment in more detail?” Sample items included “It happened with words (I was called names, yelled at, laughed at, etc.)”, “It happened physically (I was pushed, kicked and punched)”, and “It happened with the help of the Internet (rumors were spread online, videos of me were sent online against my will, etc.)”. A factor analysis indicated a one-factor solution explaining 67.16% of variance. Reliability was excellent (α = 0.92).

To differentiate between victims and bully-victims, individuals who stated that they were victimized were asked a further question concerning bullying perpetration by Burger and Bachmann [50], which had to be answered on a 7-point scale ranging from *strongly disagree* (1) to *strongly agree* (7). The wording of the item is “I myself have also teased (one or more) other classmate/s who were inferior to me for an extended period of time, with the intention of making that person suffer”.

### 2.4. Missing Data

The percentage of missing values for gender was 1.7%, for age 0.0% and for class conflict frequency 0.6%. The maximum percentage of missing values for the 28 variables measuring conflict management styles (integrating, obliging, avoiding, dominating, compromising) was 0.6%, and for the nine variables measuring psychological school adjustment 1.2%, and for the five variables measuring bullying victimization 1.7%.

### 2.5. Data Analysis

The first step was to calculate zero-order bivariate correlations between the main study variables before including them in the more complex regression-based models.

#### 2.5.1. Mediation Analysis

A multiple predictor mediation model was run using the statistics software JASP 0.14.1.0 [72]. The five different conflict management styles were included as predictors, bullying victimization as a mediator, and psychological school adjustment as an outcome, controlling for gender, age, and frequency of conflict in the classroom. A full information maximum likelihood estimator was used that could also handle missing values. Direct, indirect, total indirect, and total effects were computed.

#### 2.5.2. Moderation Analysis

A moderated regression with multiple moderator variables was computed using PROCESS [73] in IBM Statistics 27. The predictor variable was bullying victimization. Moderator variables were the five conflict management styles, and the outcome variable was school adjustment. Gender, age and general classroom conflict frequency were included as covariates. All variables that pertained to interaction terms were mean-centered in order to ease interpretability of the results. A heteroscedasticity consistent standard error and covariance matrix estimator was used (Huber-White).

#### 2.5.3. Determination of Person-Oriented Bullying-Related Groups

Using a person-oriented approach, bully-related groups were formed. In accordance with the technique used by Menesini and Camodeca [74] and Kollerová et al. [75], any individual who scored at least 0.5 *SD*s above the overall sample mean on bullying victimization was assigned to a temporary victimization group. Next, within that group, those victims were identified who disclosed that they also engaged in bullying perpetration during their school years. Finally, mutually exclusive bullying-related groups were formed, representing noninvolved individuals, pure victims, and bully-victims. Descriptive statistics and group percentages were computed for these three groups.

#### 2.5.4. Differences between Person-Oriented Bullying-Related Groups

A series of ANCOVAs was conducted to test for differences between noninvolved individuals, victims and bully-victims on different conflict management styles and psychological school adjustment. Results were controlled for gender, age and class conflict frequency.

## 3. Results

### 3.1. Zero-Order Correlations of Main Study Variables

Bivariate zero-order relations among the main study variables are displayed in Table 1. Being female was linked to higher levels of the avoiding conflict management style. A higher conflict frequency in the school class was related to being a victim and being a bully-victim, to higher levels of victimization, to lower levels of the integrating, obliging and compromising conflict management styles, and to lower levels of psychological school adjustment. Being a victim and being a bully-victim were both positively associated with victimization and negatively associated with school adjustment. Being a bully-victim was also negatively correlated with the obliging conflict management style. School adjustment was further correlated negatively with victimization and positively with the integrating and compromising conflict management style. Regarding intercorrelations between conflict management styles, integrating was positively correlated with dominating, compromising and obliging. Further, obliging was positively correlated with avoiding and compromising. Dominating was negatively correlated with obliging and avoiding.

### 3.2. Mediation Model: Bullying Victimization Mediating Effect of Conflict Management Styles on School Adjustment

**Figure 2:**
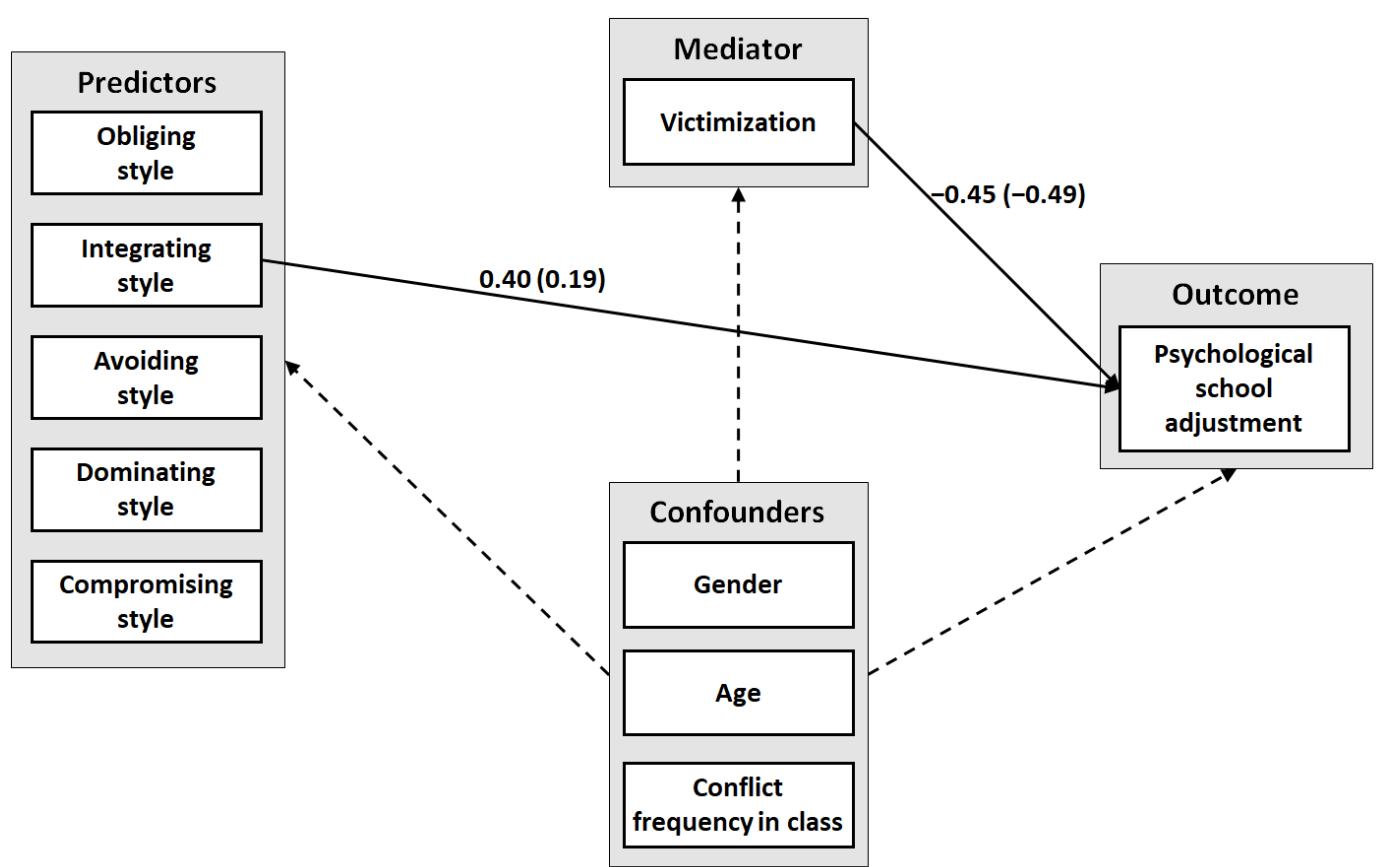
Mediation Model: The Effect of Conflict Management Styles on Psychological School Adjustment with Bullying Victimization as Mediator Variable. *Note.* Mediation analysis was computed using JASP [72]. Solid paths and path coefficients represent significant loadings (*p* ≤ 0.05). Loadings are unstandardized coefficients with standardized coefficients in parentheses.

A mediation model with multiple predictors and covariates (see Figure 2) was used to determine path coefficients effects (Supplementary Table S1) and total, direct, indirect effects (Table 2). There was a significant negative effect (γ = −0.450, *p* < 0.001) of victimization (mediator) on school adjustment (outcome), but no significant effects of conflict management styles (predictors) on victimization (mediator). Thus, no indirect effects were found. Although the model showed no significant total effects of the conflict management styles on school adjustment (however the integrating style showed a marginally significant positive effect and the avoiding style showed a marginally significant negative effect), a positive direct effect of the integrating style on school adjustment was found (γ = 0.397, *p* = 0.03). No further significant direct or indirect effects were found.

### 3.3. Moderation Model: Conflict Management Styles Moderating the Effect of Victimization on School Adjustment

A moderated regression model predicting psychological school adjustment with bullying victimization as predictor and conflict management styles as moderators, and gender, age and class conflict frequency as covariates was computed with the PROCESS macro [73] (see Table 3). All predictors and covariates defining interaction terms were mean-centered to facilitate interpretability. A heteroscedasticity consistent standard error and covariance matrix estimator was used (Huber-White). The model was significant, *F*(14,152) = 11.136, *p* < 0.001, and explained 43.29% of variance of psychological school adjustment.

A positive conditional main effect on school adjustment was found for integrating conflict management style and a negative conditional main effect was found for bullying victimization. For participants with average levels of victimization and average levels of other conflict management styles, with all other predictors being equal, the integrating conflict management style was positively associated with psychological school adjustment (*b*_integrating_ = 0.443, *p* = 0.015). For participants with average levels of conflict management styles with all other predictors being equal, victimization was negatively associated with psychological school adjustment (*b*_victimization_ = −0.464, *p* < 0.001).

**Figure 3:**
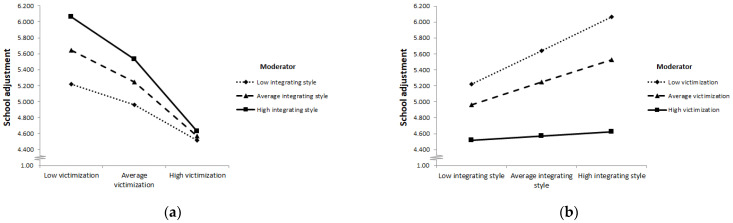
A profile plot of the ordinal interaction effect of integrating conflict management style and victimization on school adjustment. (**a**) Psychological school adjustment (unstandardized score plotted on the vertical axis) as a function of victimization (low, average, high; plotted on the horizontal axis) for different levels of integrating conflict management style (low, average, high; plotted as separate lines). (**b**) Psychological school adjustment (unstandardized score plotted on the vertical axis) as a function of integrating conflict management style (low, average, high; plotted on the horizontal axis) for different levels of victimization (low, average, high; plotted as separate lines). *Note.* Data for visualizing the conditional effects were taken from the syntax output of the PROCESS macro v4.00 [73]. Values for victimization (low = 1.00; average = 1.86; high = 3.31); values for integrating conflict management style (low = 3.30; average = 3.95; high = 4.59).

Furthermore, the effect of victimization on school adjustment was significantly moderated by the integrating conflict management style. The inclusion of the interaction term between victimization and integrating conflict management style significantly accounted for a further 1.5% of explained variance, *F*(1,152) = 3.896, *p* = 0.050. The Johnson-Neyman technique for probing conditional effects for different values of the moderator integrating conflict management style showed that, for participants with average and high levels of the integrating style (92.22% of participants), victimization was negatively associated with school adjustment, whereas there were no significant association between victimization and school adjustment for participants with low levels of integrating conflict management style (below the score of 3.01; 7.78% of participants; see Supplementary Table S2). The reason for the non-significant relationship between victimization and school adjustment at low integrating style is that students already had low school adjustment scores at low and average victimization. A visual representation of the interaction effect of integrating style is shown in Figure 3. Individuals with low integrating style have low scores in school adjustment relatively independently from victimization. For persons with medium or high integrating style, school adjustment is still relatively high at low or medium levels of victimizations but drops to a low level of school adjustment (to about the same level as for persons with low integrating style) at high levels of victimization. In other words, the integrating style does not predict school adjustment at high levels of victimization.

### 3.4. Person-Oriented Bullying-Related Groups

Adopting the approach followed by Menesini and Camodeca [74] and Kollerová et al. [75], all individuals who were at least 0.5 *SD*s above the overall sample mean in bullying victimization were allocated into a temporary victimization group (*n* = 44). Next, victims were identified who reported also having committed bullying perpetration (bully-victims; *n* = 21). Finally, the following mutually exclusive bullying-related groups were formed (see Table 4): (1) 74.0% noninvolved individuals, (2) 13.6% victims, and (3) 12.4% bully-victims. These three groups neither differed significantly in the percentage of women nor in their average age. Noninvolved individuals (*M*_noninvolved_ = 2.74, *SE* = 0.08) reported lower levels of classroom conflict frequency than both victims (*M*_victims_ = 3.39, *SE* = 0.18, *M*_diff_ = 0.66, *SE*_diff_ = 0.20, *t* [2] = 3.33, *p*_tukey_ = 0.003, Cohen’s *d* = 0.78) and bully-victims (*M*_bully-victims_ = 3.43, *SE* = 0.19, *M*_diff_ = 0.69, *SE*_diff_ = 0.21, *t* [2] =3.39, *p*_tukey_ = 0.003, Cohen’s *d* = 0.84).

### 3.5. Person-Oriented Bulling-Related Group Differences in Conflict Management Styles and Psychological School Adjustment

**Figure 4:**
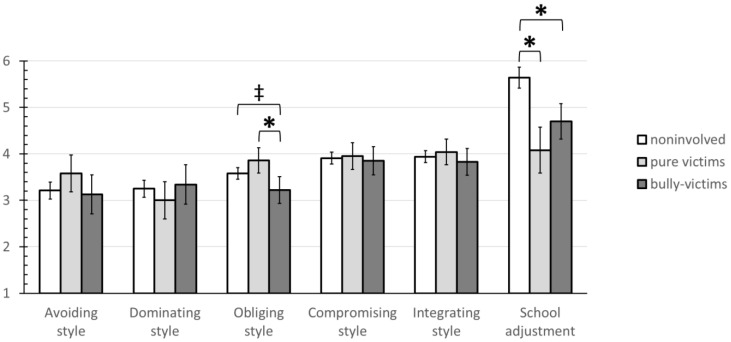
ANCOVA Results: Adjusted Means of Person-Oriented Bullying-Related Groups Regarding Conflict Management Styles and Psychological School Adjustment. *Note.* Marginal mean estimates are unstandardized scores adjusted for gender, age, and conflict frequency in class. Conflict management style is measured on a 5-point scale and psychological school adjustment on a 7-point scale. Whiskers represent 95% confidence intervals. Results of Tukey post-hoc tests: ‡ *p* ≤ 0.10, * *p* ≤ 0.05 (including lower significance levels).

A series of ANCOVAs (see Table 5 and Figure 4) revealed a significant effect of bullying-related groups on the *obliging* conflict management style and on *psychological school adjustment* after controlling for gender, age, and conflict frequency in class. No significant multivariate effects between the three groups were found for *integrating*, *avoiding*, *dominating*, and *compromising* conflict management styles.

Tukey post-hoc tests comparing the three bullying-related roles on the *obliging style* revealed significant mean differences between bully-victims (*M*_adj_ = 3.22, *SE* = 0.15) and victims (*M*_adj_ = 3.86, *SE* = 0.14; *M*_diff_ = 0.64; *SE*_diff_ = 0.19, *t* [2] = 3.31, *p* = 0.003, *d* = 0.74). Furthermore, there was a marginally significant difference between bully-victims (*M*_adj_ = 3.22, *SE* = 0.15) and noninvolved individuals (*M*_adj_ = 3.58, *SE* = 0.06; *M*_diff_ = 0.36; *SE*_diff_ = 0.16, *t* [2] = 2.30, *p* = 0.058, *d* = 0.60). Moreover, Tukey post-hoc tests on *psychological school adjustment* revealed significant mean differences between noninvolved students (*M*_adj_ = 5.64, *SE* = 0.12) and both victims (*M*_adj_ = 4.08, *SE* = 0.25; *M*_diff_ = 1.56; *SE*_diff_ = 0.27, *t* [2] = 5.82, *p* < 0.001, *d* = 1.44) and bully-victims (*M*_adj_ = 4.70, *SE* = 0.27; *M*_diff_ = 0.95; *SE*_diff_ = 0.28, *t* [2] = 3.38, *p* = 0.003, *d* = 0.84).

## 4. Discussion

It is sometimes argued that the way students handle conflict can protect against or lead to future bullying victimization. Furthermore, different conflict management styles can enhance or mitigate the negative impact of ongoing victimization on psychological school adjustment. So far, it has never been empirically tested whether different conflict management styles actually can make a difference in whether students become victimized and in how victimization affects their school adjustment. The current research project has conducted this long outstanding study. In addition to a mediation model that tested whether different styles of conflict management are associated with victimization which in turn might lead to negative school adjustment, a moderation model was calculated to test whether the use of different conflict management styles can alleviate or strengthen the negative association of victimization on school adjustment. These variable-oriented analyses were complemented by a person-oriented approach comparing victims, bully-victims and noninvolved students with regard to conflict management styles and psychological school adjustment to gain a deeper insight into what sets these groups apart.

For the generation of research hypotheses, three conceptual views of bullying victimization were contrasted: (1) the *bullying as normal conflict* view, which regards bullying as a conflict [38,39,40,41] and hypothesizes that adaptive conflict management styles, compared to maladaptive conflict management styles, should lead to less victimization and reduce the negative impact of victimization on school adjustment, (2) the *bullying as escalated conflict* view, which proposes a gradual development from conflict to bullying victimization [1,43,44] and hypothesizes that victimization and its negative effects are promoted by escalating styles and inhibited by de-escalating styles, and (3) the *bullying being distinct from conflict* view which regards bullying as qualitatively different from conflict [4,6,45] and hypothesizes that even adaptive conflict management styles have no positive effect when used in a bullying context.

The most important findings of this study are that the data are fully consistent with the hypotheses of the *bullying being distinct from conflict* view and inconsistent with the hypotheses derived from the other two views. None of the conflict management styles were related to bullying victimization and the styles also failed to dampen or intensify the negative effect of victimization on school adjustment. The person-oriented group comparison showed that it is useful to distinguish between pure victims and bully-victims because victims are using higher levels of the obliging style than bully-victims. Still, both victims and bully-victims showed lower levels of psychological school adjustment than noninvolved individuals.

### 4.1. The Integrating Style Positively Impacts Psychological School Adjustment

While in the bivariate zero-order analysis both the integrating and compromising styles had a positive effect on school adjustment, in the multivariate models only the integrating conflict management style showed a positive effect on school adjustment. All other conflict styles had no significant effects. This is in line with research that considers cooperative and active styles to be adaptive because they ensure that both one’s own and the others’ interests are respected in finding solutions to conflicts [27]. It is interesting to note that the less cooperative and passive styles (e.g., dominating, avoiding, obliging), which are generally considered to be more maladaptive [28,30,31,32], did not negatively affect school adjustment. These results could be explained by the fact that the nature of the effects of the particular styles depends on the context of the situation [23,27,33].

### 4.2. Conflict Management Styles Neither Protect against Nor Lead to Victimization

The assumption that bullying either is a conflict or begins as conflict that gradually escalates, implies that adaptive (i.e., integrating, compromising) or de-escalating (i.e., avoiding, obliging) conflict management styles act as protective barriers against victimization and that maladaptive (i.e., dominating, avoiding, obliging) or escalating styles (i.e., dominating) act as gateways to victimization. However, the mediation analysis showed that none of the conflict management styles had an effect on victimization, and thus did not have an indirect effect on school adjustment via the victimization pathway.

This confirms findings that situational factors [76] such as the behavior of the counterpart and especially the power differential [77] between both parties play a major role when it comes to the usefulness of conflict management styles. It has been proposed that conflict management styles would not predict actual behavior when the perpetrator is more powerful and aims to harm the counterpart instead of finding a solution [46]. In such situations, the availability and meaningful applicability of conflict management styles are severely limited [1,59]. Adaptive collaborative strategies such as compromising and integrating require that both parties work together constructively and non-hierarchically. Attempting to reach a compromise or even to dominate would require highly assertive behavior from the weaker person, which is not only unrealistic and ineffective [44] but would come with a high risk of challenging the perpetrator to reinforce the victimization or retaliate against the victim. In the school context, the victims usually do not have the possibility to avoid the perpetrators, since the victims cannot simply change class or school, and perpetrators can actively seek them out in order to bully them. The only option left to the victims is the obliging style, and the degree to which they submit or resist to using this submissive style (lower or higher obliging) may be related to whether they are victims or bully-victims. In summary, the reason why conflict management styles fail is that bullying is a structural problem, and thus far out of the scope of individual victim strategies [62].

The results of the present study are inconsistent with previous findings from studies in the workplace context, where the dominating style was associated with higher bullying rates and problem-solving styles were associated with lower bullying rates [56,57,58]. However, this apparent contradiction can be resolved upon closer examination of the study designs, as these studies did not examine the conflict management styles of the victims, but rather focused on conflict management styles at the team or institutional level. Since in the case of bullying there is a clear power advantage for perpetrators, it can be assumed that the perpetrators can implement their preferred conflict management styles in the team setting while the victims fail to do so. The conflict management styles at the team level therefore most likely reflect the styles used by the perpetrators and not those of the victims. The different results can thus be explained by the fact that the present study focuses on predicting victimization rather than perpetration [44].

### 4.3. Conflict Management Styles Neither Reinforce Nor Attenuate the Negative Effects of Victimization on School Adjustment

If one accepts that conflict management styles cannot prevent victimization in power-imbalanced situations, one might still hope that the use of conflict management styles might at least represent a way for victims to better handle ongoing victimization. The idea would be that different conflict management styles can mitigate (or intensify) the negative effects of victimization on psychological school adjustment. However, the results of this study show that this is not the case. An interesting result is that while higher levels of the integrating style led to better school adjustment at low levels of victimization, this positive effect decreased with increasing victimization and disappeared completely at high levels of victimization. This clearly shows that at high victimization the structural conditions either prevent the application of the preferred style (here integrating) or make it ineffective. This is consistent with previous findings that the perception of limitations, or the perception that one’s counterpart is not positively disposed toward one, causes victims to feel weaker, abandon their usual strategies and regress to a lower level of conflict management styles that are less effective [1,2,33,46,59]. It is clear that the integrating style, which aims to fulfill the interests of both sides completely, is difficult to implement when the other party sabotages the solution finding process. The cognitive load of finding an optimal solution under these impossible circumstances would lead to psychological stress [28] and bind a large amount of cognitive and emotional resources that would be lacking in other areas.

The results of this study are not consistent with previous findings in the workplace setting that cooperative conflict management styles may lead to poorer psychological outcomes when used in ongoing bullying victimization [61,62]. They are also not in line with the results of a study conducted in a Chinese school setting [23]. These conflicting results may be explained by critical differences between the school and the work environment and by the fact that in the more collectivist Chinese culture, the expectations and consequences of different conflict management styles might be different from those in the more individualistic Western countries [63,78].

### 4.4. Bullying Victimization Is Qualitatively Different from Normal Peer Conflict

The results of this study confirms that bullying is not a normal conflict [4], but a form of continued, intended and systematic abuse of power. Normal peer conflict is an inevitable part of everyday school life and is often associated with positive outcomes and the acquisition of social skills [79]. While the occasional conflict gone wrong can be harmful (e.g., when two parties using a variety of non-adversarial strategies cannot find a solution to their conflict [80]), bullying is inherently and intentionally malicious because it is created by the perpetrator with the purpose of harming the other person. Therefore, conflict management is not appropriate for preventing bullying victimization in the first place, nor is it appropriate for stopping ongoing victimization or the negative effects of ongoing victimization on school adjustment. However, this is not to suggest that conflict management in general is not important. Being able to deal with normal conflict constructively leads to positive peer relations and more peaceful behavior [81]. This is the reason why some anti-bullying programs also foster adequate conflict management and resolution skills among students [3,82,83,84]. These elements should never be stand-alone but complementary. Since the dynamic of bullying victimization is driven almost exclusively by a more powerful perpetrator acting on a less powerful victim, it does not seem appropriate to consider the less powerful victim as having the ability to improve the situation [46]. It is also known that in most cases when students are victimized, other students are present and watch but do not intervene, in part because of fear of becoming victims themselves [85]. It is therefore clear that it takes someone who is not part of this power dynamic among peers to intervene [86]. Teachers are ideally suited for this task because they already know the students, are the adults who spend the most time with students, and have a legal obligation to protect students from harm [87].

### 4.5. Distinguishing between Victims and Bully-Victims Brings Additional Insights

The results of this study also make it clear that it is useful and contributes to a more accurate understanding if a distinction is made between pure victims and bully-victims. The person-oriented group comparisons showed that while victims use higher levels of obliging style, bully-victims use lower levels of obliging style. This is consistent with findings in previous studies that victims are more likely to use accommodating and placating behaviors in the hope that the victimization will disappear if they do nothing to antagonize the perpetrators [17]. Bully-victims, on the other hand, seem to show their resistance in exhibiting less obliging behavior. Furthermore, both victims and bully-victims had lower school adjustment scores than noninvolved students. This corroborates the variable-oriented results that bullying victimization has negative effects on school adjustment and is in line with previous research [50,71].

### 4.6. Theoretical Implications

It is interesting to note that the effects of conflict management styles have been researched almost exclusively in the area of workplace bullying. This study expanded the literature by connecting this research tradition to the research tradition of school bullying. It is clear that research on school bullying can benefit from theories available from workplace bullying research. However, it must be kept in mind that the school and the workplace are completely different contexts in many ways. Not only do the institutional contexts differ, but also the maturity, tasks and behavioral motives of the actors. Future research should look more closely at what aspects the two bullying domains share, where exactly there are differences, and how these differences affect behavioral dynamics. It is worth noting, for example, that much of the school bullying research assumes that the perpetrator’s power advantage already exists a priori, whereas much of the workspace bullying research assumes that this power advantage develops over time [88].

### 4.7. Practical Implications

This study underscores the importance that researchers, policymakers, school principals, and teachers should not label bullying victimization as conflict. In cases of bullying, even students with good conflict management skills may be helpless and unable to break out of the vicious peer dynamic on their own. Schools must therefore ensure that they protect those affected by bullying. The school leadership must also be aware that they can do more harm than good by conflating incidents of peer conflict with bullying cases, and vice versa [40]. A strategy aimed solely at resolving a conflict would not be appropriate if the conflict is actually bullying, as these two types of circumstances require totally different types of interventions. In cases of bullying, effective intervention by teachers who are outside the peer class dynamic (i.e., third party intervention [89]) is optimally indicated [87] (although it might be helpful to empower the other students in the class to intervene as a complementing strategy [90]). It is important that teachers recognize bullying and take responsibility by intervening and setting clear boundaries for perpetrators, making it clear that their behavior is unacceptable and will not be allowed to continue [86]. It makes sense for teachers to seek help from other colleagues and, if indicated, to consult with or involve specialists (e.g., counselors, school psychologists). It is also important that teachers raise the issue of bullying and its serious consequences in class discussions [87]. Mediation, in which teachers try to act as an impartial between the victims and perpetrators, is not appropriate in bullying victimization [46]. Apart from bullying situations, schools should also support students to resolve conflicts in an open and collaborative way, because the ability to integrate diverging positions improves the school climate in the longer term and is a necessary condition for maintaining democratic systems as a society [91].

### 4.8. Limitations and Future Directions

In addition to notable strengths such as examining both mediated and moderated effects, combining variable- and person-oriented approaches, and bringing together research on workplace and school bullying, the current study also has some limitations. The data in the study were collected at one point in time, so assumed cause-and-effect relationships can only be based on theoretical considerations. It cannot be excluded that the relationships of the variables are more complex in reality (e.g., bidirectional relationships or self-reinforcing feedback loops). Furthermore, the results are based on a German-speaking sample of young adults with a relatively high level of education who reported retrospectively on their experiences during their school years [50]. It cannot be ruled out that participants’ reported retrospective experiences are influenced in part by their memory bias or selective memory. Caution must be exercised in making generalizations to other cultures and age groups [12,20,92]. Moreover, because bullying generally affects only a fraction of students, the victim and bull-victim groups were small. Future research should replicate the findings in a larger sample in other cultures and in other age groups and should also investigate potential gender effects. Larger samples would also allow smaller effects to be found when testing for mediation and moderation effects.

## 5. Conclusions

The implications of these findings are highly significant for bullying research, as they show that conflict management styles are not able to prevent or promote the occurrence of bullying victimization, nor are they able to reduce or increase negative effects of bullying on school adjustment. It is important that researchers, policymakers, school principals, and teachers do not label bullying as conflict. Successfully stopping bullying victimization and its negative effects requires the involvement of adults who stand outside the student bullying dynamic. Teachers are an obvious choice here, as they are the adults who spend the most time with students in school and are also legally obligated to protect students from harm.

## Figures and Tables

**Table 1:** Descriptive Statistics and Bivariate Zero-Order Correlations of the Main Study Variables.

Variable	*M*	*SD*	01	02	03	04	05	06	07	08	09	10	11	12
01. Gender (0 = *female*)	0.23	–	—											
02. Age	22.63	2.13	−0.04	—										
03. Class conflict frequency	2.91	0.91	0.03	−0.02	—									
04. Pure victim (0 = *no*)	0.14	–	−0.0003	−0.11	0.21 **	—								
05. Bully−victim (0 = *no*)	0.12	–	−0.02	0.02	0.21 **	−0.15 ‡	—							
06. Victimization	1.89	1.47	−0.03	−0.1	0.43 ***	0.61 ***	0.56 ***	*0.92*						
07. Integrating CM style	3.92	0.68	−0.004	−0.05	−0.25 **	0.01	−0.12	−0.14 ‡	*0.85*					
08. Obliging CM style	3.57	0.67	−0.02	0.03	−0.21 **	0.14 ‡	−0.23 **	−0.12	0.40 ***	*0.80*				
09. Avoiding CM style	3.33	0.94	−0.15 *	−0.05	−0.11	0.14 ‡	−0.07	0.02	−0.08	0.43 ***	*0.84*			
10. Dominating CM style	3.13	0.95	0.11	0.01	0.15 ‡	−0.09	0.09	0.04	0.23 **	−0.26 ***	−0.38 ***	*0.84*		
11. Compromising CM style	3.88	0.68	0.04	−0.05	−0.17 *	0.00	−0.07	−0.13 ‡	0.67 ***	0.43 ***	0.05	0.07	*0.74*	
12. Psych. school adjustment	5.24	1.35	0.06	0.09	−0.37 ***	−0.41 ***	−0.24 **	−0.57 ***	0.28 ***	0.06	−0.14 ‡	−0.01	0.21 **	*0.93*

*Note.* CM = conflict management; significant values (*p* ≤ 0.05) are formatted in bold; if applicable, internal consistency reliabilities (Cronbach alphas) are displayed in italics in the main diagonal if applicable. ‡ *p* ≤ 0.10, * *p* ≤ 0.05, ** *p* ≤ 0.01, *** *p* ≤ 0.001.

**Table 2:** Total, Direct, Total Indirect and Indirect Effects of Victimization Mediating the Association Between Conflict Management Styles and Psychological School Adjustment.

							95% CI		
Predictors	Mediator	Outcome	Estimate	*SE*	*z*	*p*	Lower	Upper	*Std* (*nox*)
**Total effects**									
Integrating	—	School adjustment	0.346 ‡	0.209	1.661	0.097	−0.062	0.755	0.165 ‡
Obliging	—	School adjustment	−0.132	0.183	−0.718	0.473	−0.491	0.228	−0.064
Avoiding	—	School adjustment	−0.213 ‡	0.122	−1.738	0.082	−0.453	0.027	−0.147 ‡
Dominating	—	School adjustment	−0.115	0.116	−0.991	0.322	−0.343	0.113	−0.080
Compromising	—	School adjustment	0.166	0.188	0.883	0.377	−0.203	0.534	0.081
**Direct effects**									
Integrating	—	School adjustment	0.397 *	0.181	2.186	0.029	0.041	0.752	0.189 *
Obliging	—	School adjustment	−0.195	0.160	−1.222	0.222	−0.508	0.118	−0.095
Avoiding	—	School adjustment	−0.161	0.107	−1.513	0.130	−0.370	0.048	−0.111
Dominating	—	School adjustment	−0.129	0.101	−1.269	0.204	−0.327	0.070	−0.089
Compromising	—	School adjustment	0.098	0.164	0.597	0.551	−0.223	0.418	0.048
**Indirect effects**									
Integrating	Victimization	School adjustment	−0.050	0.104	−0.483	0.629	−0.253	0.153	−0.024
Obliging	Victimization	School adjustment	0.063	0.091	0.693	0.488	−0.116	0.243	0.031
Avoiding	Victimization	School adjustment	−0.052	0.061	−0.844	0.399	−0.171	0.068	−0.036
Dominating	Victimization	School adjustment	0.013	0.058	0.230	0.818	−0.101	0.127	0.009
Compromising	Victimization	School adjustment	0.068	0.094	0.730	0.466	−0.115	0.252	0.033

*Note.* Mediation analysis was computed using JASP [72]. Delta method standard errors; full information maximum likelihood estimator. Significant estimates (*p* ≤ 0.05) are formatted in bold. ‡ *p* ≤ 0.10, * *p* ≤ 0.05.

**Table 3:** The Effect of Victimization on School Adjustment Moderated by Conflict Management Styles while Controlling for Gender, Age and Class Conflict Frequency.

	Effect on SchoolAdjustment	*SE*	*t* Value	*p* Value	Lower Level 95% *CI*	Upper Level 95% *CI*
**Intercept**	4.931 ***	0.843	5.849	<0.001	3.265	6.597
**Confounders**						
Gender	0.139	0.159	0.875	0.383	−0.175	0.454
Age	0.039	0.035	1.104	0.271	−0.031	0.109
Class conflict frequency	−0.200 ‡	0.117	−1.705	0.090	−0.432	0.032
**Conditional main effects**						
Victimization	−0.464 ***	0.073	−6.372	<0.001	−0.608	−0.320
Integrating style	0.443 *	0.181	2.456	0.015	0.087	0.800
Obliging style	−0.211	0.146	−1.446	0.150	−0.500	0.077
Avoiding style	−0.149	0.107	−1.401	0.163	−0.360	0.061
Dominating style	−0.096	0.101	−0.952	0.343	−0.297	0.104
Compromising style	0.099	0.174	0.568	0.571	−0.245	0.443
**Interaction terms**						
Victimization*integrating style	−0.245 *	0.124	−1.974	0.050	−0.490	0.000
Victimization*obliging style	−0.064	0.103	−0.621	0.536	−0.269	0.140
Victimization*avoiding style	0.064	0.082	0.786	0.433	−0.097	0.225
Victimization*dominating style	−0.014	0.078	−0.182	0.856	−0.168	0.140
Victimization*compromising style	0.206	0.139	1.480	0.141	−0.069	0.481

*Note.* The PROCESS Macro [73] (model 1) was used with psychological school adjustment as dependent variable (*y*), victimization as the focal predictor (*x*), integrating style as moderator (*W*), and the following covariates: gender, age, class conflict frequency, obliging style, avoiding style, dominating style, compromising style, interaction victimization*integrating style, interaction victimization*obliging style, interaction victimization*avoiding style, interaction victimization*dominating style and interaction victimization*compromising style. All predictors and covariates that define interaction terms were mean-centered to ease interpretability (means of conflict management styles: *M*_integrating_ = 3.92, *M*_obliging_ = 3.57, *M*_avoiding_ = 3.33, *M*_dominating_ = 3.13, *M*_compromising_ = 3.88). A heteroscedasticity consistent standard error and covariance matrix estimator was used (Huber-White). Significant vales (*p* ≤ 0.05) are formatted in bold. ‡ *p* ≤ 0.10, * *p* ≤ 0.05, *** *p* ≤ 0.001.

**Table 4:** Demographic Description of the Person-Oriented Bullying-Related Groups.

	Noninvolved	Pure Victims	Bully-Victims	Comparison Results	
**Frequency comparison**				Χ^2^(2)	
Sample size (%)	125 (74.0%)	23 (13.6%)	21 (12.4%)	125.586 ***	
Number of females (%)	96 (76.8%)	17 (73.9%)	16 (76.2%)	0.100	
**Mean comparison**				*F*(2,166)	η_p_^2^
Age (*SD*)	22.72 (2.16)	22.04 (2.08)	22.71 (2.05)	0.993	0.012
Frequency of conflicts in class (*SD*)	2.74 (0.78)	3.39 (1.12)	3.43 (1.03)	9.807 ***	0.106

*Note.* Significant values (*p* ≤ 0.05) are formatted in bold. *** *p* ≤ 0.001.

**Table 5:** ANCOVA Results: Adjusted Means and Standard Errors in Person-Oriented Bullying-Related Groups Regarding Conflict Management Styles and Psychological Adjustment in School.

	Noninvolved		Pure Victims		Bully-Victims		ANCOVA Results	
Conflict Management Styles and School Adjustment	*M*_adj_	*SE*	*M*_adj_	*SE*	*M*_adj_	*SE*	*F*(2, 161)	η_p_^2^
Integrating style	3.94	0.065	4.04	0.141	3.83	0.148	0.592	0.008
Obliging style	3.58	0.064	3.86 *c*	0.140	3.22 *b*	0.147	5.500 **	0.064
Avoiding style	3.21	0.094	3.58	0.203	3.13	0.214	1.738	0.021
Dominating style	3.25	0.094	3.00	0.205	3.34	0.216	0.877	0.011
Compromising style	3.91	0.067	3.95	0.147	3.85	0.154	0.214	0.003
Psychological school adjustment	5.64 *bc*	0.116	4.08 *a*	0.252	4.70 *a*	0.265	19.641 ***	0.196

*Note.* Computed with JASP [72]. Marginal mean estimates are adjusted for gender, age, and conflict frequency in class. Significant values (*p* ≤ 0.05) are formatted in bold. The following alphanumeric indices formatted in italics indicate significant differences (at least *p* < 0.05) from the following groups: *a* noninvolved individual, *b* pure victims, *c* bully-victims. ** *p* ≤ 0.01, *** *p* ≤ 0.001.

## Data Availability

The data presented in this study are available on request from the corresponding author.

## Supplementary Materials

The following supporting information can be downloaded at: https://www.mdpi.com/article/10.3390/ijerph191811809/s1, Table S1: Parameter Estimates of the Model with Victimization Mediating the Association Between Conflict Management Styles and Psychological School Adjustment, Table S2: Integrating Conflict Management Style: Conditional Effect of the Focal Predictor Victimization on School Adjustment.
