# Supplementary material for: School Bullying Is Not a Conflict: The Interplay between Conflict Management Styles, Bullying Victimization and Psychological School Adjustment
Source: Int J Environ Res Public Health. 2022 Sep 19;19(18):11809. doi: 10.3390/ijerph191811809 (PMC9517642; doi:10.3390/ijerph191811809)
Supplement: Supplementary file 1 [file ijerph-19-11809-s001.zip › ijerph-1900777-supplementary.pdf]

International Journal of
Environmental Research and Public Health

MDPI

Supplementary Materials

School bullying is not a conflict: The interplay between conflict management styles, bullying victimization and psychological school adjustment

Christoph Burger

Supplementary Table S1. Parameter Estimates of the Model with Victimization Mediating the Association Between Conflict Management Styles and Psychological School Adjustment.

Predictors	Outcome	Estimate	*SE*	*z* Value	*p* Value	95%-*CI* Lower	95%-*CI* Upper	*Std* (all)	*Std* (nox)
School adjustment	Victimization	**−0.450*****	0.061	−7.367	< 0.001	−0.569	−0.33	−0.488	−0.488
School adjustment	Integrating	**0.397***	0.181	2.186	0.029	0.041	0.752	0.189	0.189
School adjustment	Obliging	−0.195	0.160	−1.222	0.222	−0.508	0.118	−0.095	−0.095
School adjustment	Avoiding	−0.161	0.107	−1.513	0.130	−0.370	0.048	−0.111	−0.111
School adjustment	Dominating	−0.129	0.101	−1.269	0.204	−0.327	0.070	−0.089	−0.089
School adjustment	Compromising	0.098	0.164	0.597	0.551	−0.223	0.418	0.048	0.048
Victimization	Integrating	0.111	0.230	0.484	0.628	−0.340	0.563	0.049	0.049
Victimization	Obliging	−0.141	0.202	−0.697	0.486	−0.538	0.256	−0.063	−0.063
Victimization	Avoiding	0.115	0.135	0.849	0.396	−0.150	0.379	0.073	0.073
Victimization	Dominating	−0.030	0.129	−0.231	0.818	−0.283	0.224	−0.019	−0.019
Victimization	Compromising	−0.152	0.207	−0.733	0.464	−0.558	0.254	−0.068	−0.068
Integrating	Age	−0.021	0.022	−0.957	0.338	−0.065	0.022	−0.072	−0.033
Integrating	Class conflict frequency	**−0.173*****	0.053	−3.248	0.001	−0.278	−0.069	−0.243	−0.271
Integrating	Gender	−0.020	0.114	−0.177	0.860	−0.244	0.203	−0.013	−0.032
Obliging	Age	0.008	0.023	0.331	0.741	−0.037	0.052	0.025	0.012
Obliging	Class conflict frequency	**−0.170****	0.055	−3.110	0.002	−0.277	−0.063	−0.233	−0.261
Obliging	Gender	−0.036	0.117	−0.310	0.756	−0.265	0.192	−0.023	−0.056
Avoiding	Age	−0.022	0.033	−0.690	0.490	−0.086	0.041	−0.052	−0.024
Avoiding	Class conflict frequency	−0.134‡	0.078	−1.719	0.086	−0.286	0.019	−0.130	−0.145
Avoiding	Gender	**−0.355***	0.166	−2.136	0.033	−0.681	−0.029	−0.161	−0.385
Dominating	Age	−0.005	0.033	−0.149	0.881	−0.069	0.060	−0.011	−0.005
Dominating	Class conflict frequency	**0.172***	0.079	2.185	0.029	0.018	0.326	0.165	0.185
Dominating	Age	0.223	0.168	1.328	0.184	−0.106	0.552	0.101	0.240
Compromising	Class conflict frequency	−0.019	0.023	−0.810	0.418	−0.064	0.027	−0.061	−0.029
Compromising	Gender	**−0.124***	0.055	−2.242	0.025	−0.233	−0.016	−0.170	−0.190
Compromising	Gender	0.042	0.119	0.355	0.723	−0.190	0.275	0.027	0.064
Victimization	Age	−0.050	0.048	−1.037	0.300	−0.144	0.044	−0.073	−0.034
Victimization	Class conflict frequency	**0.658*****	0.121	5.449	< 0.001	0.421	0.895	0.405	0.453
Victimization	Gender	−0.117	0.248	−0.471	0.638	−0.602	0.369	−0.034	−0.080
School adjustment	Age	0.037	0.038	0.987	0.323	−0.037	0.112	0.060	0.028
School adjustment	Class conflict frequency	−0.191‡	0.103	−1.851	0.064	−0.394	0.011	−0.128	−0.143
School adjustment	Gender	0.126	0.195	0.644	0.519	−0.257	0.508	0.039	0.094

Note. Calculated with JASP[72]. Delta method standard errors; full information maximum likelihood estimator. *Std* = standardized estimates. Significant estimates ($p \leq 0.05$) are displayed in bold.
‡ $p \leq 0.10$, * $p \leq 0.05$, ** $p \leq 0.01$, *** $p \leq 0.001$

Supplementary Table S2. Integrating Conflict Management Style: Conditional Effect of the Focal Predictor Victimization on School Adjustment.

**Integrating conflict management style (moderator)**	**Conditional effects of victimization on school adjustment**	**Heteroscedasticity-consistent *SE***	***t* Value**	***p* Value**	**Lower level 95% *CI***	**Upper level 95% *CI***
1.000 (−2.945)	0.258	0.353	0.730	0.467	−0.440	0.955
1.200 (−2.745)	0.209	0.329	0.634	0.527	−0.441	0.858
1.400 (−2.545)	0.160	0.305	0.524	0.601	−0.442	0.761
1.600 (−2.345)	0.111	0.280	0.394	0.694	−0.443	0.665
1.800 (−2.145)	0.062	0.257	0.240	0.811	−0.445	0.568
2.000 (−1.945)	0.013	0.233	0.054	0.957	−0.447	0.472
2.200 (−1.745)	−0.036	0.209	−0.174	0.862	−0.450	0.377
2.400 (−1.545)	−0.085	0.186	−0.459	0.647	−0.453	0.282
2.600 (−1.345)	−0.134	0.163	−0.823	0.412	−0.457	0.188
2.800 (−1.145)	−0.183	0.141	−1.298	0.196	−0.462	0.096
3.000 (−0.945)	−0.232‡	0.120	−1.930	0.055	−0.470	0.006
3.012 (−0.933)	**−0.235****	0.119	−1.976	0.050	−0.471	0.000
3.200 (−0.745)	**−0.281****	0.101	−2.779	0.006	−0.481	−0.081
3.303 (−0.642)[1]	**−0.307*****	0.092	−3.318	0.001	−0.489	−0.124
3.400 (−0.545)	**−0.330*****	0.085	−3.882	< 0.001	−0.498	−0.162
3.600 (−0.345)	**−0.379*****	0.074	−5.131	< 0.001	−0.525	−0.233
3.800 (−0.145)	**−0.428*****	0.070	−6.105	< 0.001	−0.567	−0.290
3.945 (0.000)[2]	**−0.464*****	0.073	−6.372	< 0.001	−0.608	−0.320
4.000 (0.055)	**−0.477*****	0.075	−6.372	< 0.001	−0.625	−0.329
4.200 (0.255)	**−0.526*****	0.087	−6.064	< 0.001	−0.698	−0.355
4.400 (0.455)	**−0.575*****	0.103	−5.565	< 0.001	−0.779	−0.371
4.587 (0.642)[3]	**−0.621*****	0.121	−5.114	< 0.001	−0.861	−0.381
4.600 (0.655)	**−0.624*****	0.123	−5.085	< 0.001	−0.867	−0.382
4.800 (0.855)	**−0.673*****	0.144	−4.681	< 0.001	−0.957	−0.389
5.000 (1.055)	**−0.722*****	0.166	−4.352	< 0.001	−1.050	−0.394

Note. Numbers in brackets represent mean-centered values. The PROCESS Macro[73] (model 1) was used with a heteroscedasticity consistent standard error (Huber-White) to obtain the Johnson-Neyman output. The value 3.012 (−0.933) of the moderator variable integrating conflict management stye defines the lower boundary of the Johnson-Neyman significance region (7.78% of values below, 92.22% above). Significant vales ($p \leq 0.05$) are formatted in bold.

[1] $M - 1SD$; [2] M, [3] $M + 1SD$

‡ $p \leq 0.10$, * $p \leq 0.05$, ** $p \leq 0.01$, *** $p \leq 0.001$
